# Effect of Thermal Processing by Spray Drying on Key Ginger Compounds

**DOI:** 10.3390/metabo15060350

**Published:** 2025-05-24

**Authors:** Alina Warren-Walker, Manfred Beckmann, Alison Watson, Steffan McAllister, Amanda J. Lloyd

**Affiliations:** Department of Life Sciences, Aberystwyth University, Aberystwyth, Wales SY23 3FL, UK; arw21@aber.ac.uk (A.W.-W.); meb@aber.ac.uk (M.B.); aan@aber.ac.uk (A.W.); stm64@aber.ac.uk (S.M.)

**Keywords:** spray drying, ginger, gingerols, shogaols, metabolomics, carrier agents, functional foods, bioactive compounds, ultra-processed

## Abstract

Background/Objectives: Spray drying is a technique widely employed in the food and nutraceutical industries to convert liquid extracts into stable powders, preserving their functional properties. Ginger (*Zingiber officinale*) is rich in bioactive compounds such as gingerols, shogaols, and zingerone, which contribute to its health benefits. This study aimed to investigate the impact of spray drying on the chemical profile of ginger, particularly focusing on the transformation of gingerols into shogaols and related compounds. Methods: Fresh ginger juice was spray-dried using various carrier agents, including Clear Gum (CO03), pea protein, and inulin. Mass spectra of the resulting powders were acquired using High-Resolution Flow Infusion Electrospray Ionisation Mass Spectrometry (HR-FIE-MS) to obtain fingerprint data. Key bioactive compounds were tentatively identified to Level 2, and their relative intensities were assessed to evaluate the effects of different carriers on the chemical composition of the ginger powders. Results: Spray drying with the commercial carrier CO03 resulted in an increase in shogaol analogues ([10]-, [8]-, and cis-[8]-shogaol), gingerenone B, and oxidation products such as 6-hydroxyshogaol, 6-dehydroshogaol, and zingerone. In contrast, natural carriers like pea protein and inulin led to lower relative intensities of these bioactives, suggesting limited capacity for promoting thermal transformations. Spray drying without a carrier produced a shogaol-dominant profile but resulted in powders with poor handling properties, such as stickiness and agglomeration. Antioxidant and total polyphenol assays showed that spray drying reduced antioxidant capacity, while total polyphenol content was more preserved; natural carriers such as inulin better maintained bioactivity compared to modified starch or pea protein. Conclusions: Among the five formulations evaluated—ginger juice with no carrier, with CO03 (two dilutions), pea protein, or inulin—CO03-based samples showed the greatest chemical transformation, while inulin and pea protein better preserved antioxidant capacity but induced fewer metabolite changes. Thus, choice of carrier in the spray-drying process influences the chemical profile and functional characteristics of resultant ginger powders. While CO03 effectively enhances the formation of bioactive shogaols and related compounds, its ultra-processed nature may not align with clean-label product trends. Natural carriers, although more label-friendly, may not create the desired chemical transformations. Therefore, optimising carrier selection is important to balance bioactivity, product stability, and consumer acceptability in the development of ginger-based functional products.

## 1. Introduction

Spray drying (drying by liquid atomisation) is a thermal technique widely used in the food, beverages, and nutraceutical industries for converting liquid extracts into stable, dry powders while preserving their functional and organoleptic properties [1]. The process involves atomising a liquid feed into a heated chamber, where rapid evaporation results in the formation of a fine powder. One of the advantages of spray drying is its ability to encapsulate and preserve thermally sensitive bioactive compounds, including phenolics, flavonoids, and essential oils, maintaining the nutritional and bioactive/functional qualities of the original raw material. In addition to preserving chemical goodness, spray drying extends product shelf life and improves handling characteristics, enhancing the stability, flowability, and reconstitution properties of powders.

Ginger (*Zingiber officinale*) is a rhizome renowned for its health-promoting compounds, such as gingerols, shogaols, paradols, hydroxyshogaols, gingerenones, and zingerone. Gingerols are the major non-volatile bioactive components in ginger and are primarily responsible for its distinctive pungency. They have been reported to exhibit a wide range of pharmacological activities, including anti-inflammatory, antioxidant, and metabolic effects [2,3]. During thermal processing, 6-gingerol undergoes dehydration, where the hydroxyl group at the C-5 position is eliminated, forming a double bond between C-4 and C-5 to yield 6-shogaol, increasing the overall pungency of ginger [4,5,6]. Notably, 6-gingerol has been shown to reduce inflammation and oxidative stress and is considered a promising candidate for reducing inflammatory markers and lipid enzyme expression in high-fat diet-induced obesity models [7,8]. Similarly, shogaols, particularly 6-shogaol, are potent bisphenolic compounds with anti-inflammatory, antioxidant, and gastroprotective effects, including maintenance of intestinal mucosal integrity [7,9]. Additionally, hydroxyshogaols and gingerenones, such as 6-hydroxyshogaol and gingerenone A/B/C, are formed through oxidative and rearrangement reactions of shogaols during thermal treatment [6]. Methylated analogues like methyl-8-shogaol and methylgingerols may exhibit enhanced thermal stability, allowing them to remain stable or accumulate depending on processing conditions [6]. Other thermally influenced metabolites such as paradols and zingerone—structural analogues of gingerols—also contribute to ginger’s health benefits [2]. Zingerone has been shown to improve hepatotoxicity by regulating oxidative stress, inflammation, and apoptosis in animal models [2]. However, the presence of compounds such as zingerone may indicate loss of certain bioactivities due to degradation [4,5]. These structural changes highlight the importance of controlling temperature and processing time to change the functional (bioactivity) and sensory properties and consumer acceptability of ginger products.

Spray drying can stabilise the chemistry of bioactives in powder form, facilitating their incorporation into functional food products and supplements, as well as extending product shelf life and improving handling characteristics. Thermal processing during spray drying can cause chemical transformations, such as the dehydration of gingerols to more bioactive shogaols, as mentioned above. The selection of appropriate carrier materials during spray drying can enhance encapsulation efficiency, influence the retention of volatile chemistry, and modify the physical properties of the resulting powders. Carrier agents—such as modified starch, plant proteins, and dietary fibres—are commonly used to aid drying efficiency, protect bioactives, and influence the physicochemical properties of the final powder, with natural carriers increasingly favoured for clean-label formulations. Spray drying can act as a preservation and functional enhancement method, producing ginger-based functional ingredients with extended usability and optimised bioactive content [10,11]. 

To monitor chemical changes during food and drink processing, metabolomic fingerprinting has emerged as a powerful analytical approach [12,13]. By enabling comprehensive fingerprinting of low molecular weight metabolites, metabolomics provides detailed insights into the biochemical and bioactive transformations that occur under different thermal (such as spray drying), enzymatic, or mechanical treatments [14,15,16]. In the context of ginger processing, fingerprinting can be used to observe near-global chemical changes in products and help in the identification of key transformation products, such as the conversion of gingerols into shogaols and the formation of oxidation products like gingerenones and zingerone [13]. Therefore, metabolomics represents a critical tool for the design and quality control of processed ginger products and other functional food ingredients, aiding in optimising conditions for the preservation or enhancement of health-promoting chemistry.

This study aimed to investigate the impact of spray drying on the bioactive composition of ginger. By employing metabolomic fingerprinting, we monitored the chemical transformations that occurred during the spray-drying process and with different carriers. Additionally, the research evaluated the functional properties of the resulting ginger powders, including their antioxidant activity and total polyphenol content, to determine their potential efficacy in health-promoting applications. The overall aim was to optimise spray-drying conditions to preserve or enhance ginger’s bioactive compounds, contributing to the development of high-quality ginger-based nutraceutical products.

## 2. Materials and Methods

### 2.1. Ginger Preparation

Fresh ginger rhizomes used in this study were grown by Adam yn yr Ardd, a Welsh horticulturist that supports community-based, sustainable agriculture. All ginger was cultivated in Wales in non-heated greenhouses, harvested at full maturity, and stored in cool, dry conditions before processing. For processing, the rhizomes were washed under running water to remove surface debris. Peeling was not performed to preserve the outer epidermis. The ginger was chopped into 1–2 cm segments, then processed in a Kenwood Multipro Classic food processor for approximately 3 min to achieve a coarse pulp. The pulp was transferred to a sterile muslin cloth and manually pressed over a bowl to extract the juice. The extraction was repeated until the majority of liquid was removed. The extracted juice underwent low-temperature pasteurisation using the high-temperature short-time (HTST) method, heated to 72 °C for 15 s to reduce microbial load while preserving flavour and phytochemical content. The pasteurised juice was stored in sterilised, food-grade containers at 4 °C.

The residual ginger pulp was evenly spread on trays and dehydrated using a Buffalo Dehydrator, CS950 (Nisbets, Bristol, UK) at 50 °C for 12 h, or until moisture content was reduced below 10%. The dried pulp was then sealed in airtight containers and stored at room temperature.

### 2.2. Spray Drying

#### 2.2.1. Materials

Spray drying was performed using a European Lab ESDT1 Spray Dryer (European Spray Dry Technologies Ltd., Essex, UK). The dryer has an evaporation capacity of approximately 1.5 L/h. The ESDT1 is designed for research and educational applications, offering precise control over drying parameters and efficient processing of heat-sensitive materials.

The spray-drying assembly comprised an aspirator that maintained negative pressure and directed the drying airflow through the unit, a two-fluid nozzle for atomising the liquid feed into fine droplets, and a vertical drying chamber where atomised droplets were rapidly dried upon contact with hot air. The drying chamber was constructed from borosilicate glass, allowing clear observation of the drying process, and was designed to minimise wall deposition and ensure efficient powder separation.

The two-fluid nozzle used had an orifice diameter of 0.5 mm, facilitating fine droplet formation and consistent powder morphology. The feed solution was delivered using an integral peristaltic pump at a controlled flow rate, with atomisation air pressure maintained to ensure efficient dispersion into the drying chamber. Drying air was supplied through an adjustable heater unit, with inlet temperatures ranging from 120 °C to 220 °C, and outlet temperatures monitored and maintained between 80 °C and 90 °C, depending on the formulation. The system included a cyclone separator for powder collection, minimising product loss and enabling accurate yield determination. All process parameters were monitored digitally, and system performance remained stable across all trials.

Fresh ginger juice, containing 4.92% total soluble solids, was used as the core feed material. The dry matter content of the feed was determined with a halogenic drier MB35 (OHAUS Europe GmbH, Nönikon, Switzerland) at 120 °C until no change in mass occurred anymore. A range of carrier agents were employed to aid encapsulation and improve powder characteristics, including modified starch ((Clear Gum CO03, Rooquette, Lestrem, France), pea protein (Roquette, France), and inulin (Detox Trading, Devon, UK)). Deionised water was used to dilute formulations to appropriate concentrations for atomisation. All materials were food grade and used without further purification.

#### 2.2.2. Drying Parameters

Five formulations were prepared to investigate the influence of carrier type and dilution on powder recovery and stability. One formulation (SD_pure) consisted of ginger juice alone and served as a control. The remaining formulations included one of the selected carriers at specified concentrations, as outlined in Table 1. Carrier concentrations and added water volumes were varied to optimise feed viscosity and drying behaviour, while the ginger juice mass was kept constant at 137 g across all carrier-containing formulations.

Spray drying was carried out under varying inlet and outlet air temperature conditions tailored to each formulation. Inlet temperatures ranged from 160 °C to 180 °C, while outlet temperatures were maintained between 80 °C and 90 °C, depending on the formulation.

Ginger juice containing 4.92% solids was used across all formulations. Five formulations were prepared (one with no carrier), with the details of compositions and spray drying conditions summarised in Table 1.

### 2.3. Sample Preparation for Analysis

Dry ginger powders were extracted following a Bligh and Dyer protocol [17,18,19]. Approximately 5 mg of each powdered sample was weighed into triplicate Eppendorf tubes. To each tube, 1 mL of extraction solution (water:methanol:chloroform, 2:5:2, *v*/*v*/*v*) was added. Samples were vortexed for 10 s, then shaken on a Kühner shaker at 4 °C and 140 rpm for 30 min. Following a second 10 s vortex, the samples were sonicated in an ice-chilled water bath for 15 min, and then frozen overnight at −80 °C. Storage in the freezer of the liquid Bligh and Dyer extraction mix is part of the extraction process to remove proteins quantitatively.

After freezing, samples were again vortexed briefly and centrifuged at 13,000 rpm for 6 min at 4 °C. From each supernatant, 100 µL was transferred to a Liquid Chromatography–Mass Spectrometry (LC-MS) vial containing an insert, and 50 µL was pooled into a new Eppendorf tube labelled master mix (MM) for quality control and matrix matching. The pooled MM sample was thoroughly mixed and centrifuged under the same conditions. Two 200 µL aliquots of the MM were transferred into fresh vials with inserts for analytical quality control injections. A control blank containing only the extraction solution (2:5:2) was prepared and processed alongside the experimental samples. Vials were capped and labelled with the corresponding sample IDs and vial numbers for traceability.

For the preparation of liquid ginger juice samples, frozen ginger juice samples were thawed at room temperature, vortexed thoroughly for 20 s, and 500 µL aliquots were transferred into labelled Eppendorf tubes. To each aliquot, 500 µL of chilled PT-methanol was added. Tubes were frozen overnight at −80 °C in preparation for solvent removal via SpeedVac the following day. After drying, the contents were weighed to determine dry matter yield, and approximately 5 mg of the dried juice was scraped into new tubes for extraction using the same procedure described for dry powders. Based on a calculated dry matter content of ~25 mg/mL, an equivalent 100 µL aliquot of juice was taken for standardisation. This was extracted by adding 100 µL H_2_O, 500 µL MeOH, and 200 µL CHCl_3_, and then processed according to the dry sample extraction protocol above (starting with the vortexing step). Figure S1 shows a graphical scheme of sample preparation workflow for metabolomics.

### 2.4. Antioxidants and Total Polyphenols

All dry samples were stored at 4 °C prior to analysis. The juice sample was stored at −20 °C and thawed overnight at 4 °C before processing. The samples were extracted using a 2:1 ratio of solvent to sample, using 1000 µL of acidified 70% methanol solution (methanol: ultrapure water: 1 M HCl 70:29.5:0.5) [20]. For the juice sample, two 500 µL aliquots were transferred to 2 mL Eppendorf tubes. Each aliquot was extracted with 1 mL of acidified 70% methanol. Samples were vortex-mixed and then shaken at 700 rpm at 25 °C in the dark for 1 h on a rotary shaker. After extraction, precipitates were removed by centrifugation at 10,000× *g* for 10 min at 4 °C. Supernatants were transferred to fresh Eppendorf tubes and stored on ice prior to analysis. For the dried powder samples, duplicate extracts were prepared by weighing 100 mg of each powder into 2 mL Eppendorf tubes and adding 1 mL of acidified 70% methanol. The same extraction, shaking, centrifugation, and storage procedures were followed as for the liquid sample.

Total antioxidant capacity was measured using the Sigma-Aldrich Antioxidant Assay Kit (Merck, Rahway, NJ, USA, catalogue number MAK334), according to the manufacturer’s instructions. This kit measured total antioxidant capacity in which Cu^2+^ is reduced by an antioxidant to Cu^+^, which forms a coloured complex with a dye reagent. The assay was carried out in flat-bottom 96-well plates using 20 µL aliquots of each sample in triplicate. The assay reaction consisted of adding 100 µL of freshly prepared reaction mix from the kit and incubating with gentle shaking at room temperature for 10 min. The colour intensity measured at 570 nm (using an Agilent BioTek Epoch absorbance microplate spectrophotometer) was proportional to the total antioxidant capacity in the sample. Blank values (A_570_ of 20 µL of ultrapure water) were subtracted from all test samples. The kit supplied the synthetic vitamin E analogue Trolox (6-hydroxy-2,5,7,8-tetramethylchroman-2-carboxylic acid) as the standard, which gave a linear detection range of 1.5–1000 µM. Samples exceeding the linear range of the standard curve were diluted with ultrapure water, and the results were corrected by the appropriate dilution factor. The total antioxidant capacity (TAC) of the samples was expressed as µM Trolox equivalents.

Total phenolic compounds were quantified using the Sigma-Aldrich Phenolic Compounds Assay Kit (Merck, catalogue number MAK365), following the manufacturer’s protocol. In this assay, phenolic compounds coupled with diazonium salts under alkaline conditions to form a stable diazo chromophore, detectable by absorbance at 480 nm. This microplate assay used the flavan-3-ol Catechin as the standard, which gave a linear response between 2 and 10 nmole/well. For each reaction well, 50 µL of sample or diluted standard was mixed with 80 µL of buffer and 20 µL of probe, before incubating with gentle shaking at room temperature for 10 min before measuring the absorbance at 480 nm (A_480_). Samples were run in triplicate, along with their background controls, which consisted of 50 µL of sample and 100 µL of buffer only. The A_480_ values of the background controls were subtracted from the A_480_ values of each sample, before comparing the background-corrected sample A_480_ values to the Catechin standard curve to obtain the nmole of product (diazo chromophore) generated during the reaction. Samples exceeding the linear range of the standard curve were diluted with ultrapure water as needed. The following calculation was used to determine the mM Catechin equivalents in each sample: Sample phenolic compound concentration (mM or nmol/µL Catechin equivalents).

### 2.5. High-Resolution Flow Infusion Electrospray Ionisation Mass Spectrometry (HR-FIE-MS)

While FIE-ESI-MS does not include chromatographic separation, it provides a rapid and reproducible means of acquiring high-resolution metabolic fingerprints across large sample sets. Mass spectra were acquired on a QExactive Plus (Thermo Finnigan, San Jose, CA, USA) mass spectrometer, coupled with a Dionex UltiMate 3000 ultra-performance liquid chromatography (UHPLC) system (Thermo-Scientific, Waltham, MA, USA). Metabolite fingerprints were generated in both positive and negative ionisation modes, in a single run.

All samples were randomised to minimise batch effects using a computer-generated randomisation sequence and also to account for potential matrix effects from carrier agents. This ensured that different samples were evenly distributed across analytical runs. Samples (20 µL) were injected into a flow of 100 µL min^−1^ methanol:water (70:30, *v*/*v*). Quality control (QC) samples were prepared by pooling aliquots from all sample extracts, and these were injected at regular intervals during each sequence to monitor instrument performance, drift, and ionisation consistency. Ion intensities were acquired between *m*/*z* 55 and 1200 for 3.5 min at a resolution setting of 120,000, resulting in 3 (±1) ppm mass accuracy. Tuning and ESI source parameters were set according to the manufacturer’s recommendations. Further details are found in [12,21,22,23,24,25].

### 2.6. Data Analysis

Following data acquisition, Chromeleon.cmbx files were first exported to .raw files and then converted to the .mzML open file format and centroided [26] using msconvert (Trans Proteomic Pipeline) [27]. Spectral binning was applied using the R package binneR [28], and then standard post-acquisition processing routines were applied, including occupancy and QC filtering. Data were normalised and log2 transformed. Representative total ion count (TIC) chromatograms of the infusion profile of the samples (negative and positive mode) and averaged mass spectrum fingerprints (negative and positive mode) are shown in Figure S2. Putative molecular formulas were generated by using MZedDB [29], an Aberystwyth University database for accurate mass annotation to 3 (±1) ppm accuracy. The ionisation products of the assigned molecular formulas were first searched against the KEGG compound database for putative matches. Initial data analysis including classification was performed in R package metabolyseR. Features were identified to Level 2, as defined by the Metabolomics Standards Initiative (MSI) [30].

Principal Component Analysis (PCA) was performed using the *prcomp* function in R [31]. Supervised Random Forest (RF) classification was implemented using the randomForest package in R [31]. For all Random Forest models, the number of trees (*ntree*) used was 1000 and the number of variables considered at each internal node (mtry) was the square root of the total number of variables. Unsupervised RF classification models were plotted following Multi-Dimensional Scaling (MDS). Proximity measures for each individual observation were extracted from RF models and scaled coordinates produced using *cmdscale* on 1-proximity. Top-ranked features contributing to the MDS models were extracted using re-sampling methods and *p*-values of False Positive Rates (FPR <= 0.05).

## 3. Results

### 3.1. Spray Drying Parameters

Spray drying was conducted under the conditions described in Section 2.2. The key physical and processing characteristics of the resulting ginger powders are summarised below.

Modified starch (Clear Gum CO03) was selected for its known film-forming and encapsulation capabilities. In the formulation SD_CO03_478, ginger juice and modified starch were combined with 478 g of water and spray-dried at an inlet temperature of 160 °C and outlet temperature of 80 °C. This process yielded a fine, free-flowing powder with a final moisture content of 2.3%. The powder exhibited good handling properties, with low cohesiveness and rapid solubility, indicating successful encapsulation and drying.

In the formulation SD_CO03_800, the water addition was increased to 800 g while maintaining the same carrier-to-ginger juice ratio. Despite the higher inlet temperature of 180 °C and outlet temperature of 90 °C, the resulting powder displayed larger, more agglomerated particles, higher moisture content (4.1%), and noticeable wall deposition within the drying chamber. These observations indicate that excessive water content negatively impacted drying efficiency and powder morphology.

Pea protein (moisture content 4.4%) was incorporated as a natural, protein-rich carrier in the SD_Pea formulation. However, the formulation exhibited excessive viscosity during processing, which affected atomisation and spray-drying performance. As a result, very little powder was obtained, and significant drying inefficiency was observed due to pump blockage and feed instability.

In contrast, the SD_Inulin formulation (moisture content 2.1%), which utilised inulin at a 2:1 ratio relative to ginger solids, successfully produced a stable powder. Inulin’s water-binding capacity contributed to effective drying and powder formation, although the resulting powder demonstrated slightly lower flowability compared to the CO03-based formulations.

Moisture content was not determined for the spray-dried pure ginger juice sample due to insufficient powder yield for analysis (a non-viable commercial option).

The ginger content in the final powders was calculated based on the solid content of the ginger juice. Powders formulated with modified starch exhibited the lowest ginger concentrations (~4.7%), due to the high proportion of carrier relative to ginger solids. In contrast, powders produced with pea protein or inulin alone contained higher ginger contents (~25–33%), reflecting the lower carrier load or higher ginger solids ratio. These differences in formulation composition directly influenced the functional and physical attributes of the final products.

### 3.2. Metabolomics

**Figure 1:**
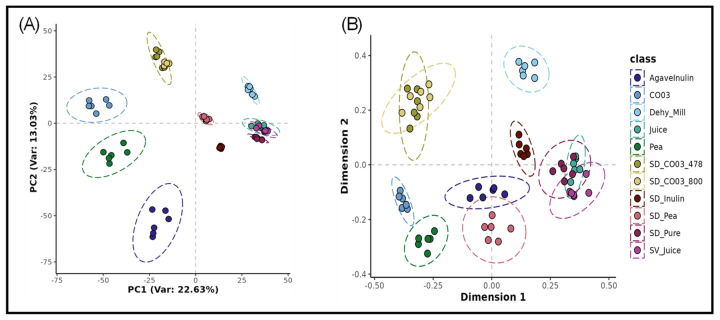
Unsupervised Principal Component Analysis (PCA) (**A**) and Multidimensional Scaling (MDS) (**B**) based on Random Forest (RF) proximity values applied to Flow Injection Electrospray–High Resolution Mass Spectrometry (FIE-HRMS) chemical metabolomic fingerprint data from ginger samples and carriers. *Where Dehy_mill, Ginger dehydrated and milled; Juice, Ginger Juice; SV_juice, speed vac ginger juice; SD_Pure, Spray Dried Pure Ginger Juice; SD_CO03_478, Spray Dried Ginger Juice, Clear Gum CO03 1:1 478 mL Water; SD_CO03_800, Spray Dried Ginger Juice, Clear Gum CO03 1:1 800 mL Water; CO03, Cleargum CO03; SD_pea, Spray Dried Ginger Juice, Pea protein; Pea, Pea Protein; SD_Inulin, Spray Dried Ginger Juice, Inulin 3:1; AgaveInulin, Agave Inulin*.

The unsupervised PCA and MDS plots (Figure 1A,B) illustrate the clustering of samples based on their ‘near global’ metabolomic fingerprints. The closeness of individual clusters reflects intra-class similarity, whereas the spatial separation between clusters represents inter-class dissimilarity. This visualisation enables the identification of natural groupings and potential chemistry that differentiates the different groups. Ginger juice (Juice), spray-dried juice without a carrier (SD_Pure), and speed-vacuum concentrated juice (SV_Juice) formed tight, well-separated clusters, indicative of distinct but related metabolic fingerprints. The dehydrated ginger pulp (Dehy_Mill) was positioned close to these groups but did not overlap, suggesting a chemically similar yet distinct profile. The three carrier materials (CO03, Pea, and Inulin) formed clusters that were metabolically distinct from all ginger-derived materials, as expected, and from each other. Next, ginger juice spray-dried with each of these mentioned carriers resulted in samples that were metabolically different from both the unprocessed juice and the carrier controls. Among these, SD_CO03_478 and SD_CO03_800 were similar to each other, forming a partially overlapping cluster, suggesting consistent formulation effects.

**Figure 2:**
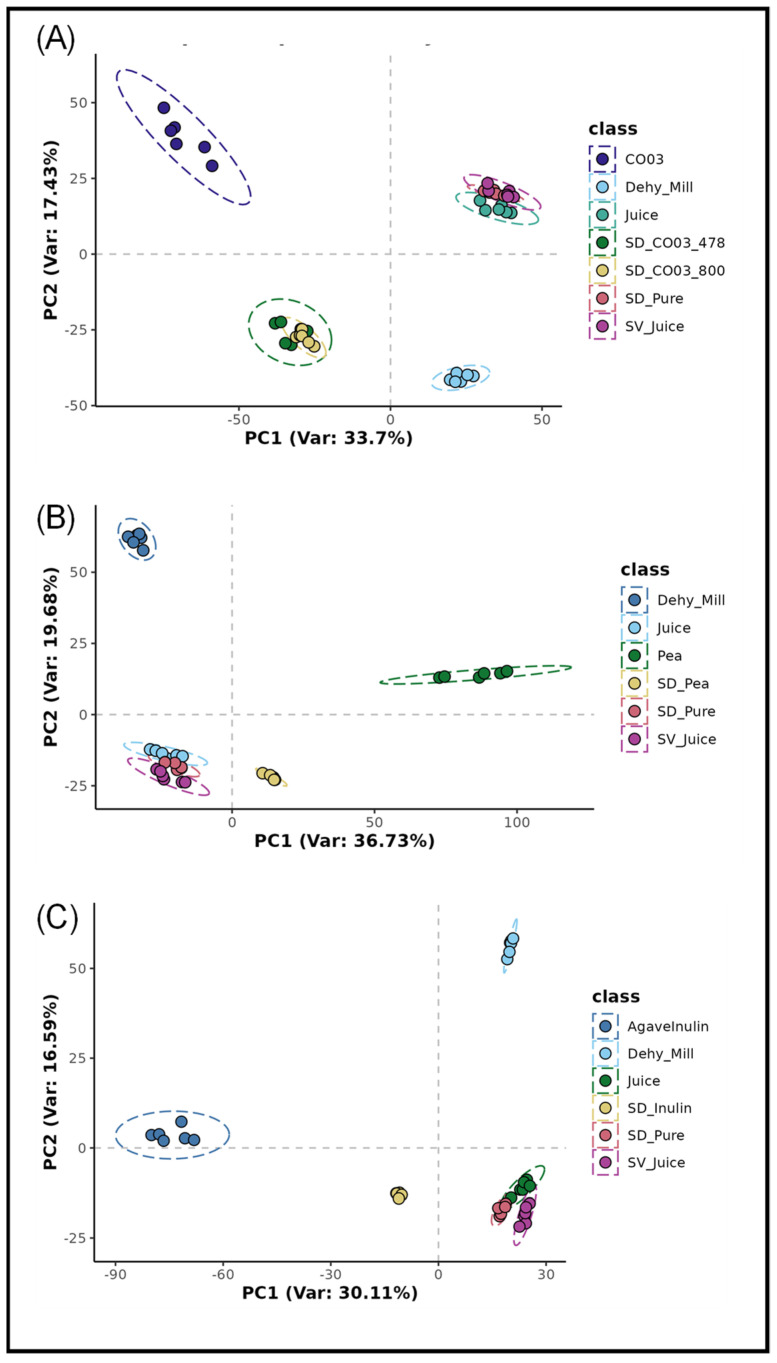
Unsupervised Principal Component Analysis (PCA) applied to Flow Injection Electrospray–High Resolution Mass Spectrometry (FIE-HRMS) chemical metabolomic fingerprint data from ginger samples and carriers (**A**) CO03; (**B**) Pea protein; (**C**) Inulin. *Where Dehy_mill, Ginger dehydrated and milled; Juice, Ginger Juice; SV_juice, speed vac ginger juice; SD_Pure, Spray Dried Pure Ginger Juice; SD_CO03_478, Spray Dried Ginger Juice, Clear Gum CO03 1:1 478 mL Water; SD_CO03_800, Spray Dried Ginger Juice, Clear Gum CO03 1:1 800 mL Water; CO03, Cleargum CO03; SD_pea, Spray Dried Ginger Juice, Pea protein; Pea, Pea Protein; SD_Inulin, Spray Dried Ginger Juice, Inulin 3:1; AgaveInulin, Agave Inulin*.

By looking at each carrier group—CO03, pea protein, and inulin—individually, a more detailed interpretation of metabolomic changes can be seen. Ginger juice spray-dried with CO03 at two temperatures (SD_CO03_478 and SD_CO03_800) showed a high degree of overlap, indicating consistent and highly similar metabolic fingerprints, despite the amount of water added and temperature parameters, as expected. Notably, both formulations were metabolically distinct from the CO03 carrier alone, suggesting that the inclusion of ginger and the spray-drying process caused specific metabolic changes. SD_CO03_478 and SD_CO03_800 also differed markedly from the Juice, SV_Juice, and SD_Juice samples, highlighting the substantial influence of the CO03 carrier on the final metabolomic profile, despite all treatments coming from the same juice input (Figure 2A).

In the pea protein treatment group, SD_Pea and Pea (carrier) clustered relatively closely together, suggesting that the incorporation of ginger juice did not greatly alter the overall chemical profile. However, when other treatments—CO03 and inulin—were removed from the plot, clearer differences emerged between SD_Pea and Pea, indicating that spray drying may still induce metabolomic changes (Figure 2B). Both samples remained clearly distinct from the Juice, SV_Juice, and SD_Juice clusters. A similar pattern was observed in the inulin group: SD_Inulin and Inulin exhibited high similarity to each other and closely mirrored the response seen with the pea formulations (Figure 2C), suggesting reduced chemical transformation during spray drying when these natural carriers were used.

**Figure 3:**
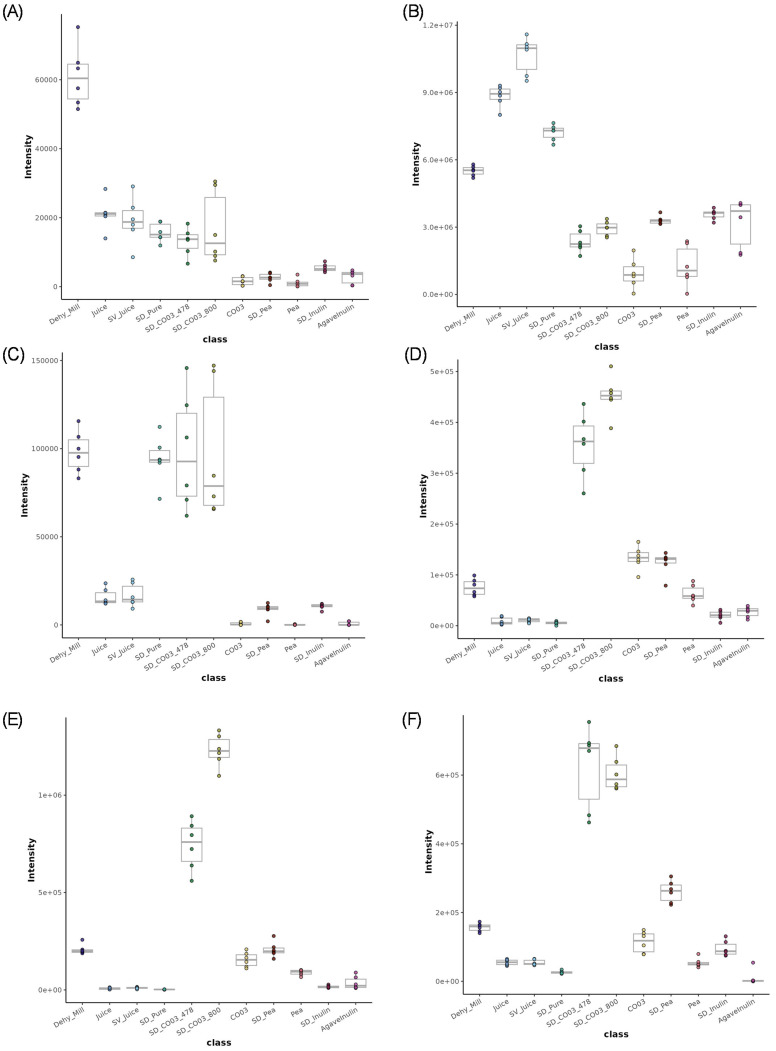
Box plots. (**A**) [12]-Gingerol; (**B**) Gingerenone B; (**C**) 10-shogaol; (**D**) 6-Hydroxyshogaol; (**E**) [6]-Dehydroshogaol; (**F**) Zingerone, where median = central line, box = interquartile range (IQR) (25th–75th percentile), whiskers = 1.5 × IQR, dots = outliers. *Where Dehy_mill, Ginger dehydrated and milled; Juice, Ginger Juice; SV_juice, speed vac ginger juice; SD_Pure, Spray Dried Pure Ginger Juice; SD_CO03_478, Spray Dried Ginger Juice, Clear Gum CO03 1:1 478 mL Water; SD_CO03_800, Spray Dried Ginger Juice, Clear Gum CO03 1:1 800 mL Water; CO03, Cleargum CO03; SD_pea, Spray Dried Ginger Juice, Pea protein; Pea, Pea Protein; SD_Inulin, Spray Dried Ginger Juice, Inulin 3:1; AgaveInulin, Agave Inulin*.

Key ginger bioactives were tentatively identified to Level 2 [30], as shown in Table S1, and their relative intensities were visualised using box plots to observe the changes after thermal processing (Figure 3A–F). This approach allowed for the assessment of how compounds such as gingerols, shogaols, and zingerone were affected by heat treatment (spray drying). The highest intensity of [12]-gingerol was observed in the Dehy_Mill (dehydrated and milled ginger) samples (Figure 3A). Juice, SV_Juice, SD_Pure, SD_CO03_478, and SD_CO03_800 samples exhibited medium levels, while intensities decreased markedly in the other spray-dried samples with pea and inulin carriers. Gingerenone B showed elevated intensities in the Dehy_Mill, Juice, SD_Pure, and SV_Juice samples compared to other groups (Figure 3B). Decreases were seen in spray-dried powders (a medium level) and baseline in the carriers. An increase in 10-shogaol was seen in Dehy_Mill, SD_Pure, SD_CO03_478, and SD_CO03_800 powders, indicating the thermal conversion of gingerols during spray drying. Samples without carrier material (SD_Pure) had the highest levels, followed by CO03-based powders (Figure 3C). Natural carriers such as pea protein and inulin resulted in lower intensities, comparable to unprocessed juice samples. 6-Hydroxyshogaol was elevated in the spray-dried powders with CO03 carriers, suggesting considerable thermal oxidation of shogaols during spray drying (Figure 3D). Other carrier-based formulations and control materials exhibited lower levels. A similar pattern was seen for [6]-Dehydroshogaol, where the spray-dried samples with CO03 showed the highest relative intensities, suggesting that high-temperature processing promotes oxidative degradation and conversion of gingerols to shogaol derivatives with certain carrier materials (Figure 3E). Zingerone levels were also elevated in CO03 samples, indicating thermal decomposition of gingerols. Spray-dried samples containing natural carriers (SD_pea and SD_Inulin) exhibited medium zingerone intensities, suggesting less thermal degradation compared to CO03 (Figure 3F).

### 3.3. Antioxidants

The antioxidant capacity and total phenolic content of the ginger juice, spray-dried powders, and carrier materials are summarised in Table 2. Dehydrated and milled ginger (Dehy_mill) exhibited the highest antioxidant capacity (1936.25 µmol Trolox equivalents) and a high total phenolic concentration (0.693 mM). Fresh ginger juice (Juice) showed a moderate antioxidant capacity (696.25 µmol Trolox equivalents) with a lower phenolic concentration (0.179 mM). Spray drying without a carrier (SD_Pure) resulted in a reduction in antioxidant capacity (431.46 µmol Trolox equivalents) relative to Dehy_mill, despite exhibiting a similar total phenolic content (0.713 mM). This suggests that while the overall phenolic pool was retained, specific antioxidant-active compounds were lost or degraded during thermal processing. Formulations containing Clear Gum CO03 (SD_CO03_478 and SD_CO03_800) exhibited comparable antioxidant capacities (~377–382 µmol Trolox equivalents) and notably lower phenolic contents (~0.051–0.065 mM).

The sample produced using inulin (SD_Inulin) demonstrated a relatively higher antioxidant capacity (549.17 µmol Trolox equivalents) and moderate phenolic content (0.158 mM). In contrast, the pea protein formulation (SD_pea) exhibited the lowest antioxidant activity among the spray-dried powders (220.21 µmol Trolox equivalents) and a correspondingly low phenolic content (0.075 mM). Analysis of the pure carrier materials revealed minimal antioxidant or phenolic contents. Clear Gum CO03 and Agave Inulin showed negligible antioxidant capacities (0.00 and 18.96 µmol Trolox equivalents, respectively), while pea protein exhibited modest antioxidant activity (87.08 µmol Trolox equivalents), consistent with the previous literature [32].

Across all samples, antioxidant capacity did not strongly correlate with total phenolic content, suggesting that possible specific subclasses of phenolics or other non-phenolic antioxidant compounds were differentially affected by the spray-drying process and carrier selection.

## 4. Discussion

Spray drying has been shown to be an effective technique for both the preservation and enhancement of bioactive compounds in botanicals, particularly through the promotion of thermally induced transformations [33]. When spray drying is performed using the commercial carrier Clear Gum CO03, a marked increase in shogaol analogues (including [10]-, [8]-, and cis-[8]-shogaol), gingerenone B, and oxidation products such as 6-hydroxyshogaol, 6-dehydroshogaol, and zingerone was observed, relative to unprocessed juice. These transformations are consistent with the known thermal degradation and dehydration pathways of gingerols [34], suggesting that CO03 facilitates both dehydration and oxidative rearrangement under spray-drying conditions. However, despite its efficacy, CO03 is considered an ultra-processed ingredient, which may limit its application in clean-label formulations aimed at health-conscious consumers or natural product markets.

While CO03 effectively enhanced the thermal transformation of gingerols into more bioactive shogaols, its classification as a modified starch would likely place it within the “ultra-processed” category according to the NOVA classification system [35,36]. However, this label has been increasingly criticised for its lack of scientific rigour, as it does not account for the functional benefits or context of use [37]. In this case, CO03 played a crucial role in stabilising thermolabile compounds and promoting beneficial chemical transformations during spray drying. The term “ultra-processed” may oversimplify complex food technologies and overlook the potential of such ingredients to enhance nutritional quality [38]. Therefore, while regulatory or consumer-facing definitions may categorise CO03 as ultra-processed, a different interpretation is warranted in scientific evaluations focused on functional and bioactive outcomes.

The use of natural carriers such as pea protein and inulin [39], although attractive from a labelling and consumer perception standpoint, resulted in lower relative intensities of all measured bioactives. This suggests limited capacity for bioactive preservation or transformation during spray drying, potentially due to inadequate encapsulation efficiency, protective barrier effects that reduce thermal conversion, or physicochemical incompatibility between the ginger extract and the carrier matrix.

Spray drying without a carrier produced a shogaol-dominant profile, particularly elevating [10]-, [8]-, and cis-[8]-shogaol, along with gingerenone B. However, no increases were observed for secondary oxidation products such as 6-hydroxyshogaol, 6-dehydroshogaol, or zingerone, suggesting that the absence of a carrier may reduce the extent of oxidative reactions. While this profile is favourable from a bioactivity perspective, the resulting powder exhibited poor handling properties—such as stickiness, agglomeration, and instability—rendering it commercially impractical for large-scale applications. Spray drying impacted the antioxidant and phenolic composition of ginger powders. Dehydrated and milled ginger retained the highest antioxidant activity, whereas spray drying, particularly without a carrier, resulted in antioxidant loss despite preserving total phenolic content. Carrier selection influenced antioxidant retention: powders produced with Clear Gum CO03 exhibited reduced antioxidant activity and phenolic levels, while inulin carriers moderately preserved bioactivity. Pea protein, although inherently antioxidant-active, did not effectively protect ginger compounds during spray drying. The lack of correlation between antioxidant capacity and total phenolics suggests selective degradation of bioactive molecules.

All in all, these findings highlight that the choice of carrier plays a pivotal role in modulating both the chemical profile and functional characteristics of spray-dried ginger powders [39]. The trade-off between enhancing or preserving bioactives and achieving desirable physical and commercial attributes must be carefully balanced. Optimising carrier selection is therefore essential to maximise the therapeutic potential and market acceptability of ginger-based functional food and nutraceutical products.

## 5. Conclusions

This study demonstrates that spray drying alters the chemical profile and functional properties of ginger juice, with the choice of carrier influencing this. Modified starch (Clear Gum CO03) promoted the thermal conversion of gingerols into more bioactive shogaols, as well as oxidative products such as 6-hydroxyshogaol and zingerone. However, there was reduced antioxidant capacity and lower total phenolic content, likely due to oxidative degradation. Natural carriers such as pea protein and inulin better preserved antioxidant capacity and native phenolic content but were less effective in promoting bioactive transformation. Spray drying without a carrier maximised shogaol production but resulted in powders with poor physical properties, limiting their practical application. These findings underline the importance of optimising spray-drying parameters and carrier materials to balance bioactive enhancement, product stability, and consumer-driven clean-label requirements in the development of ginger-based functional ingredients.

## Figures and Tables

**Table 1:** Spray-drying formulations and conditions.

Recipe	Carrier Material	Ginger Juice (g)	Carrier (g)	Water Added (g)	Inlet Temp (°C)	Outlet Temp (°C)
SD_pure	None	137	N/A	100	170	88
SD_CO03_478	Modified Starch (CO03)	137	137	478	160	80
SD_CO03_800	Modified Starch (CO03)	137	137	800	180	90
SD_pea	Pea Protein	137	37	400	180	90
SD_Inulin	Inulin	137	39.36	450	170	82

**Table 2:** Antioxidant capacity and total phenolic content.

Sample Description	Antioxidant Capacity (µmol Trolox Equivalents)	Total Phenolic Content (mM)
Ginger dehydrated and milled (Dehy_mill)	1936.25	0.693
Ginger Juice (Juice)	696.25	0.179
Speed Vac Ginger Juice (SV_juice)	*not available*	*not available*
Spray-Dried Pure Ginger Juice (SD_Pure)	431.46	0.713
Spray-Dried Ginger Juice, Clear Gum CO03 1:1, 478 mL Water (SD_CO03_478)	382.29	0.065
Spray-Dried Ginger Juice, Clear Gum CO03 1:1, 800 mL Water (SD_CO03_800)	377.29	0.051
Cleargum CO03 (CO03)	0	0.012
Spray-Dried Ginger Juice, Pea Protein (SD_pea)	220.21	0.075
Pea Protein (Pea)	87.08	0.033
Spray-Dried Ginger Juice, Inulin 3:1 (SD_Inulin)	549.17	0.158
Agave Inulin (AgaveInulin)	18.96	0.007

## Data Availability

Data will be made available on request.

## Supplementary Materials

The following supporting information can be downloaded at: https://www.mdpi.com/article/10.3390/metabo15060350/s1, Figure S1: A graphical scheme of sample preparation workflow for metabolomics. Figure S2: Total ion count (TIC) chromatograms of the infusion profile of the samples (negative and positive mode), averaged mass spectrum fingerprint (negative and positive mode). Table S1: Key ginger bioactives were tentatively identified to Level 2.

## Abbreviations

The following abbreviations are used in this manuscript:

Clear gum	CO03
master mix	MM
Liquid Chromatography–Mass Spectrometry	LC-MS
Quality control	QC
High resolution Flow Infusion Electrospray Ionisation Mass Spectrometry	HR-FIE-MS
ultra-performance liquid chromatography	UHPLC
Metabolomics Standards Initiative	MSI
Principal Component Analysis	PCA
Random Forest	RF
multi-dimensional scaling	MDS
False Positive Rates	FPR
high-temperature short-time	HTST
