# Supplementary material for: Effect of Thermal Processing by Spray Drying on Key Ginger Compounds
Source: Metabolites. 2025 May 24;15(6):350. doi: 10.3390/metabo15060350 (PMC12195494; doi:10.3390/metabo15060350)
Supplement: Supplementary file 1 [file metabolites-15-00350-s001.zip › metabolites-3645369-supplementary.pdf]

Figure S1: A graphical scheme of sample preparation workflow for metabolomics.

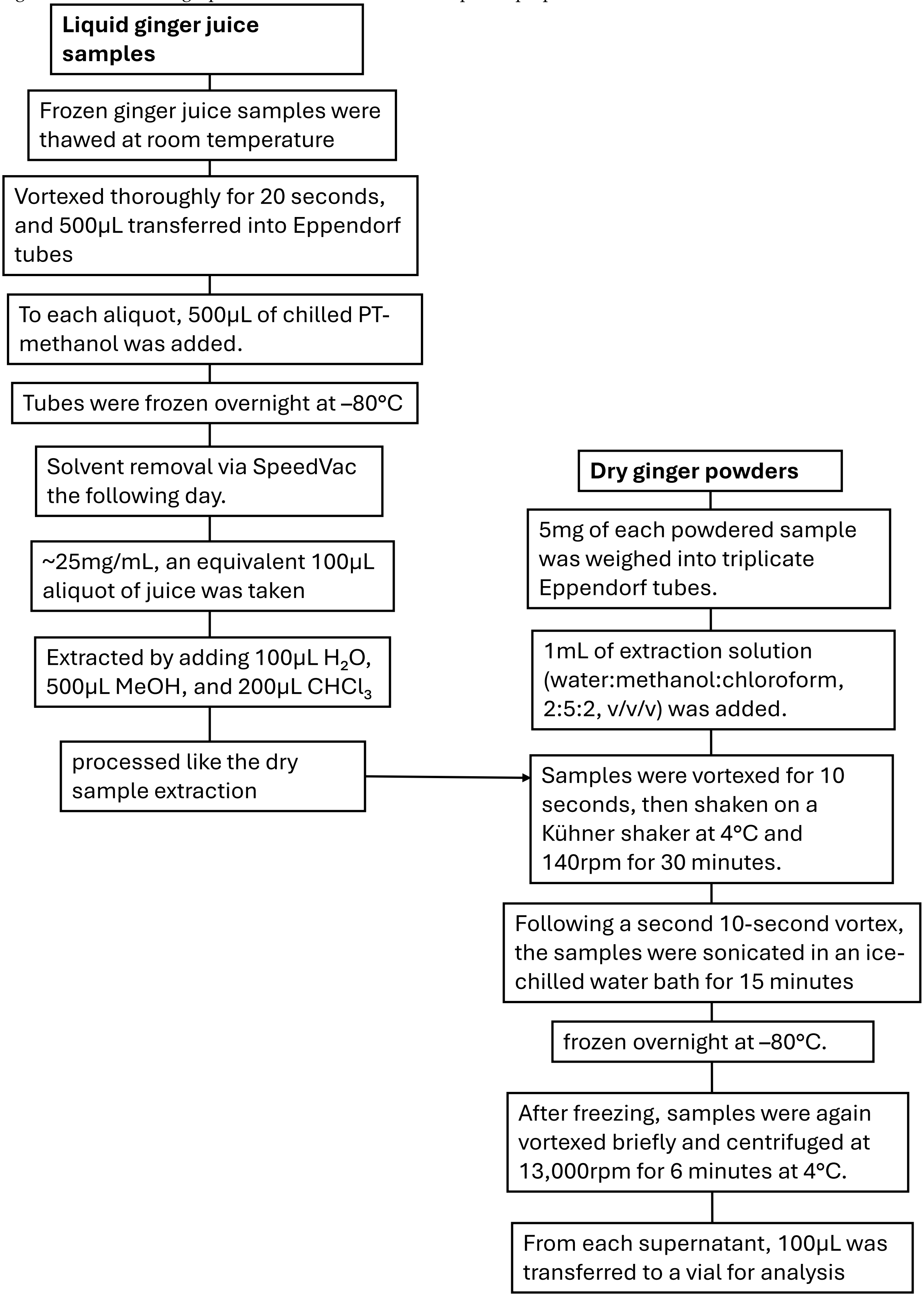

Figure S2: Total ion count (TIC) chromatograms of the infusion profile of the samples (negative and positive mode), Averaged mass spectrum fingerprint (negative and positive mode).

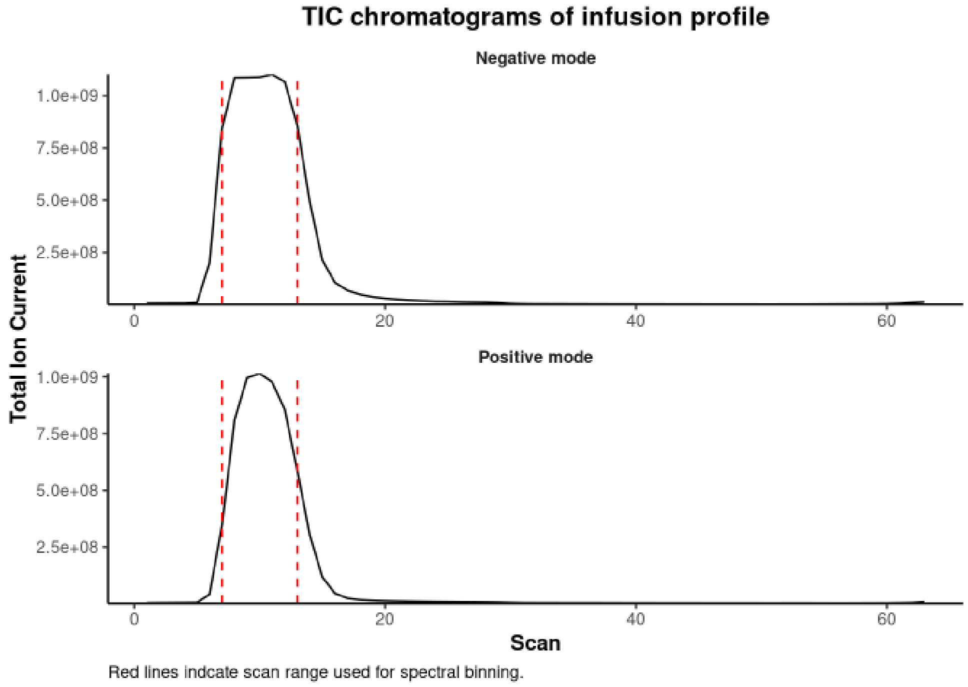

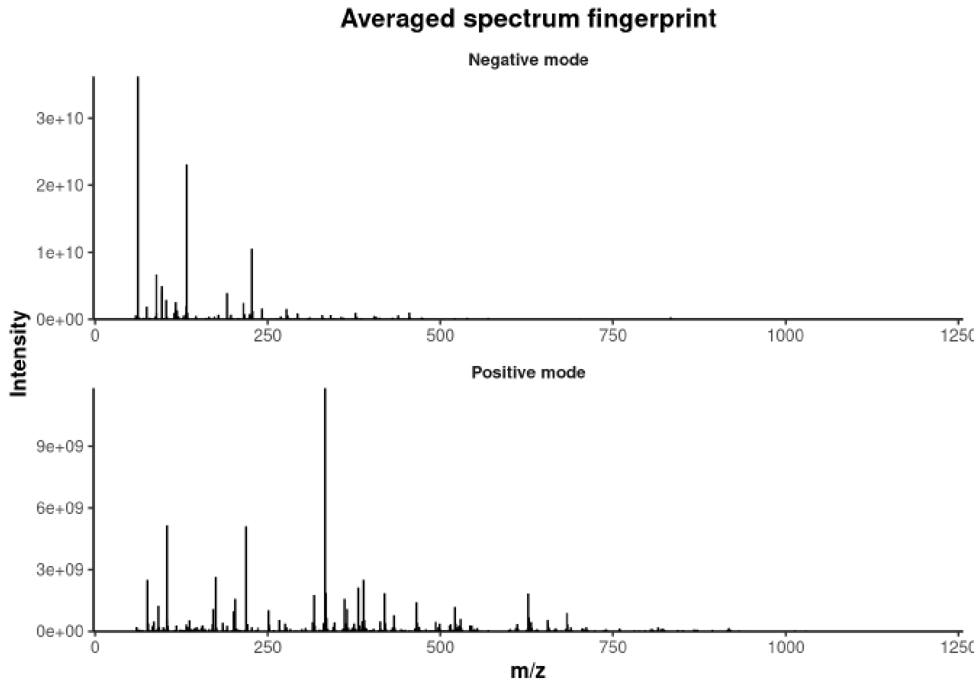

Sample TICs

Table S1: Key ginger bioactives were tentatively identified to Level 2.

NAME	MF	Adduct	Measured m/z	Theoretical m/z	PPM Error
[6]-Gingerol	C17H26O4	[M-H]1-	293.17618	293.17583	1.2
[6]-Gingerol	C17H26O4	[M+Cl]1-	329.15302	329.15251	1.5
[6]-Gingerol	C17H26O4	[M+H-H2O]1+	277.18033	277.17982	1.8
[6]-Gingerol	C17H26O4	[M+Na]1+	317.17175	317.17233	-1.8
[6]-Gingerol	C17H26O4	[M+K]1+	333.14493	333.14627	-4.0
[6]-Gingerol	C17H26O4	[M+K]1+	333.14575	333.14627	-1.6
[10]-Gingerol	C21H34O4	[M-H]1-	349.23917	349.23843	2.1
[10]-Gingerol	C21H34O4	[M+Cl]1-	385.21616	385.21511	2.7
[10]-Gingerol	C21H34O4	[M+H-H2O]1+	333.24121	333.24242	-3.6
[10]-Gingerol	C21H34O4	[M+K]1+	389.20831	389.20887	-1.4
[8]-Gingerol	C19H30O4	[M-H]1-	321.20795	321.20713	2.6
[8]-Gingerol	C19H30O4	[M+Cl]1-	357.18488	357.18381	3.0
[8]-Gingerol	C19H30O4	[M+H-H2O]1+	305.21149	305.21112	1.2
[8]-Gingerol	C19H30O4	[M+Na]1+	345.2038	345.20363	0.5
[8]-Gingerol	C19H30O4	[M+K]1+	361.17755	361.17757	-0.1
[10]-Dehydroshogaol	C21H30O3	[M+Na]1+	353.20938	353.20871	1.9
[10]-Gingerdione	C21H32O4	[M+Na]1+	371.2196	371.21928	0.9
[10]-Shogaol	C21H32O3	[M-H]1-	331.22873	331.22786	2.6
[10]-Shogaol	C21H32O3	[M+H]1+	333.24121	333.24242	-3.6
[10]-Shogaol	C21H32O3	[M+Na]1+	355.22458	355.22436	0.6
[10]-Shogaol	C21H32O3	[M+K]1+	371.19858	371.1983	0.8
[12]-Gingerol	C23H38O4	[M-H]1-	377.2706	377.26973	2.3
[12]-Gingerol	C23H38O4	[M+Cl]1-	413.24603	413.24641	-0.9
[12]-Gingerol	C23H38O4	[M+Na]1+	401.2662	401.26623	-0.1
[6]-Gingerdione	C17H24O4	[M-H]1-	291.16122	291.16018	3.6
[6]-Gingerdione	C17H24O4	[M+Cl]1-	327.13779	327.13686	2.8
[6]-Gingerdione	C17H24O4	[M+K]1+	331.1312	331.13062	1.8
[8]-Dehydroshogaol	C19H26O3	[M+Na]1+	325.17752	325.17741	0.3
[8]-Gingerdione	C19H28O4	[M+Cl]1-	355.16843	355.16816	0.8
[8]-Shogaol	C17H24O3	[M-H]1-	275.16635	275.16526	4.0
[8]-Shogaol	C17H24O3	[M+Cl]1-	311.14264	311.14194	2.2
[8]-Shogaol	C17H24O3	[M+H]1+	277.18033	277.17982	1.8
[8]-Shogaol	C17H24O3	[M+Na]1+	299.16211	299.16176	1.2
[8]-Shogaol	C17H24O3	[M+K]1+	315.13574	315.1357	0.1
6-Hydroxyshogaol	C17H24O4	[M-H]1-	291.16122	291.16018	3.6
6-Hydroxyshogaol	C17H24O4	[M+Cl]1-	327.13779	327.13686	2.8
6-Hydroxyshogaol	C17H24O4	[M+K]1+	331.1312	331.13062	1.8
cis-[8]-Shogaol	C19H28O3	[M-H]1-	303.19724	303.19656	2.2
cis-[8]-Shogaol	C19H28O3	[M+H]1+	305.21149	305.21112	1.2
cis-[8]-Shogaol	C19H28O3	[M+Na]1+	327.19339	327.19306	1.0
cis-[8]-Shogaol	C19H28O3	[M+K]1+	343.16702	343.167	0.1
Gingerenone A	C21H24O5	[M+K]1+	395.12589	395.12553	0.9
Gingerenone B	C22H26O6	[M-H]1-	385.1644	385.16566	-3.3
Gingerenone C	C20H22O4	[M+H]1+	327.15756	327.15909	-4.7
Gingerenone C	C20H22O4	[M+Na]1+	349.14081	349.14103	-0.6
Methyl-[10]-shogaol	C22H34O3	[M+Na]1+	369.2403	369.24001	0.8
Methylgingerol	C18H28O4	[M-H]1-	307.19217	307.19148	2.2

Methylgingerol	C18H28O4	[M+Cl]1-	343.1691	343.16816	2.7
Methylgingerol	C18H28O4	[M+Na]1+	331.18839	331.18798	1.2
Methylgingerol	C18H28O4	[M+K]1+	347.16193	347.16192	0.0
Zingerone	C11H14O3	[M+Na]1+	217.08369	217.08351	0.8
Zingerone	C11H14O3	[M+K]1+	233.05742	233.05745	-0.1
